# Urine biopsy technologies: Cancer and beyond

**DOI:** 10.7150/thno.44634

**Published:** 2020-06-22

**Authors:** Chun Kwan Chen, Junchen Liao, Man Sze Li, Bee Luan Khoo

**Affiliations:** Department of Biomedical Engineering, City University of Hong Kong, Hong Kong, China.

**Keywords:** Liquid biopsy, exfoliated bladder cancer cells, cell-free DNA, extracellular vesicles, disease monitoring

## Abstract

Since the discovery of circulating tumor cells in 1869, technological advances in the study of biomarkers from liquid biopsy have made it possible to diagnose disease in a less invasive way. Although blood-based liquid biopsy has been used extensively for the detection of solid tumors and immune diseases, the potential of urine-based liquid biopsy has not been fully explored. Advancements in technologies for the harvesting and analysis of biomarkers are providing new opportunities for the characterization of other disease types. Liquid biopsy markers such as exfoliated bladder cancer cells, cell-free DNA (cfDNA), and exosomes have the potential to change the nature of disease management and care, as they allow a cost-effective and convenient mode of patient monitoring throughout treatment. In this review, we addressed the advancement of research in the field of disease detection for the key liquid biopsy markers such as cancer cells, cfDNA, and exosomes, with an emphasis on urine-based liquid biopsy. First, we highlighted key technologies that were widely available and used extensively for clinical urine sample analysis. Next, we presented recent technological developments in cell and genetic research, with implications for the detection of other types of diseases, besides cancer. We then concluded with some discussions on these areas, emphasizing the role of microfluidics and artificial intelligence in advancing point-of-care applications. We believe that the benefits of urine biopsy provide diagnostic development potential, which will pave opportunities for new ways to guide treatment selections and facilitate precision disease therapies.

## Introduction

Liquid biopsy is a promising alternative to tissue biopsy in disease diagnosis and monitoring. The types of liquid biopsy samples include blood, urine, and saliva. As compared to tissue biopsy, liquid biopsy not only provides a minimal or non-invasive method for disease diagnostics and evaluation but also gives information on real-time monitoring of disease progression and therapeutic response 1. Tissue biopsy is an invasive, conditional approach (surgery depends on the health condition of patients) and is non-ideal for the long-term monitoring of patients. As compared to the standards of evaluation, such as radiologic evaluation and tissue biopsy, the liquid biopsy also has advantages in terms of compatibility for real-time detection and minimal disease detection 2. For example, radiologic evaluation methods cannot detect low counts of heterogeneous tumor cells, leading to reduced detection sensitivity, which affects early-stage disease detection.

In recent decades, the detection and characterization of such components from urine has generated much interest among the scientific and clinical communities. Methodologies associated with the isolation and characterization of urinal components are manifold and can be categorized as genomic profiling, protein profiling, and microfluidic techniques. The specificity and sensitivity of these technologies are of paramount importance, as most of the techniques require small amounts of concentrated samples for analysis 3. Microfluidic systems provide numerous benefits such as ease of operations, quick processing time, low volume sample intake, high sensitivity, and high capability of integrating functional module integration 4. The reproduction of large-scale processes remains a challenge in this field 5, 6.

Here, we discuss the advancement of research to utilise urinary components as possible markers for disease diagnosis and monitoring, including exfoliated bladder cancer cells (EBCCs), exosomes, and cell-free DNA (cfDNA). First, we focused on key diagnostic technologies widely available to the public or for use in clinical settings. Then, we shift our focus to recent technological development on cell, exosome, and cfDNA research with different disease applications. Finally, we conclude by highlighting the role of microfluidics and artificial intelligence in advancing point-of-care (POC) applications with some potential perspectives on the research of these biomarkers.

## Components of urine biopsy

Urine contains several types of cells, including epithelial cells, kidney-derived cells, white blood cells (WBC), red blood cells (RBC), and urothelial cells, as well as genetic material, proteins, peptides, and inorganic compounds. Each biomarker presents with its advantages and disadvantages, and the selection of biomarkers may depend on the type of technology available (**Table 1**). Studies have found that the cfDNA released by cells into the systemic circulation can also be filtered by the kidneys and transmitted in urine 7, 8. Therefore, scientists and clinicians are keen to utilize urine biopsies for the potential detection of diseases, including colorectal cancer (CC) 3, bladder cancer (BC) 9, chronic kidney disease 10, prostate cancer (PC) 11, cystic fibrosis 12, and chronic obstructive pulmonary disease 13.

In a urine biopsy, samples from patients are collected and analyzed to generate disease information, assist diagnosis, provide treatment direction, assess prognosis, or predict disease recurrence in real-time. Urine samples can be separated into supernatant and precipitate fractions to identify these biomarkers. For example, EBCCs, immune cells, RBC, and debris may contain tumor-derived signals for the detection and characterization of cancer 14.

### EBCCs

EBCCs are cells shed from a tumor and are found in urine. The enrichment of EBCCs can increase the sensitivity of BC detection 15. Because nucleic acids are isolated from EBCCs, genetic analysis, and fluorescence *in-situ* hybridization (FISH) methods are used to improve the accuracy of BC detection and to determine the stage of BC 16.

EBCCs in urine samples can be enriched in various ways, and the collection efficiency varies with each approach. Size-based filtration is the use of membranes to capture EBCC from clinical urine samples. It was first reported in 1979 17, and later studies have shown that filtration can improve the detection sensitivity of early and relapsed BC (87% in filtered samples and 80% in non-filtered samples) 15. Antibody-based methods have been proven to be highly selective (99%) and sensitive (100%) 18.

There are still many challenges to resolve before EBCC detection can be used in clinics. In future technological advances, other issues related to processing systems must still be addressed, such as concentrated cell viability and EBCC morphology.

### cfDNA

cfDNA can be released from healthy or diseased cells during processes that disrupt a tumor or during cell apoptosis and necrosis. Most cfDNA fragments contain 100-200 base pairs. Since phagocytes will remove cellular debris and necrotic cells under normal physiological conditions, the content of cfDNA is very low in the absence of disease 19. In the presence of disease, digestion is incomplete, DNA removal is low, and the size of DNA fragments is random (even greater than 10,000 base pairs). Therefore, the amount of cfDNA in the patient is high. Mutations in cfDNA provide vital information for diagnosis and prognosis.

cfDNA can be a useful biomarker extracted from a urine biopsy. Studies have shown that DNA molecules can penetrate the human kidney barrier after being injected into the blood and can be detected from urine samples 7. cfDNA can be detected from urine samples by ultracentrifugation procedures or using size-based selection methods to isolate total DNA by molecular weight-based fractionation 20. However, the extraction process of cfDNA presents some challenges. For example, high cfDNA fragments may evade capture, resulting in the loss or reduction of target DNA molecules. In this case, the low abundance of cfDNA can be compensated by processing a large number of samples.

The isolated cfDNA can be amplified and detected by established methods, including polymerase chain reaction (PCR), real-time polymerase chain reaction (qPCR), digital polymerase chain reaction (dPCR), and next gene sequencing (NGS). However, the frequency of detecting cfDNA mutations in urine often depends on the detection method and the source of the sample. Recent studies have shown that the concentration of cfDNA in urine is even higher than that in blood. For example, Yosuke et al. conducted gene detection tests on urine supernatants, urine precipitate, and blood samples from patients with BC, cystitis, and benign tumors 21. Half of the somatic mutations present in tumors were detected in urine samples (53% in urine supernatant and 48% in urine precipitate), while 2% were detected in the blood plasma. The diagnostic sensitivity of cancer-associated genes was 67% in urine supernatants, 78% in urine precipitate in non-invasive cancers, and in contrast to invasive cancer, 86% in urine supernatant and 71% in urine precipitate, respectively. This may be due to the presence of inflammatory cells in the urine, which reduces the purity of the target cells and reduces the detection rate of mutations because many normal alleles are contaminated in the urine sediment. Another factor will be the inherent differences between individuals, the extent to which tumor cells undergo apoptosis and necrosis, resulting in the shedding of tumor-derived DNA. Overall, the results demonstrate that urine-based urothelial BC detection is more sensitive than cytology.

The use of cfDNA as a diagnostic biomarker can also be extended to non-urological cancers such as non-small cell lung cancer (NSCLC) and CC 22. For example, T790 M resistance mutations and EGFR-activated mutations in patients are closely related to the presence of advanced NSCLC and can help assess drug resistance and patient relapse rates 23. CC-related hypermethylated *vimentin* gene and mutated *K-ras* sequences have also been found in urine 24, 25. Interestingly, cfDNA also serves as a potential biomarker for non-cancer-related diseases, as Epstein-Barr virus (EBV) DNA may be derived from urine samples and has been used as a biomarker for patients with infectious mononucleosis 26. EBV DNA has also been shown to be a non-specific biomarker for nasopharyngeal carcinoma 27. Also, in some cases, prolonged EBV activity may cause chronic active EBV disease and be a biomarker for lymphoma.

### Extracellular vesicles (EVs)

EVs are lipid bilayer particles released from cells, with diameters ranging from 30 nm to 5000 nm. The main components of EVs can be divided into exosomes, microvesicles, and apoptotic bodies 28. Despite extensive research, the size range of EVs has not been clearly defined. Among them, exosomes are endocytic EVs with a size between 30 and 100 nm, while microvesicles derive from the plasma membrane and range between 50 nm to 1000 nm 29-31. Apoptotic bodies that originate from the membrane of apoptotic cells may range from 500 nm to 5000 nm 32.

EVs can be found in various types of liquid biopsy, such as blood 33, urine 29, saliva 34, breast milk 35, and cerebrospinal fluid 36. EVs contain complex nucleic acids. Exosomes and microvesicles contain other molecules such as cytoplasmic proteins, membrane proteins, and RNA. Similarly, exosomes can transport or transfer proteins and nucleic acids to mediate cell communication 37. RNA related to EVs, such as microRNA (miRNA) and mRNA, can be transcribed and translated into DNA and protein, respectively, to affect downstream pathways. Among them, miRNAs secreted from EVs can exist in a stable state in body fluids, including urine, and play a key role in intercellular communication 38. As such, there has been a strong awareness of the potential of EVs as biomarkers in clinical practice. For example, some miRNA levels are significantly higher in early urothelial carcinoma and can be used as biomarkers for cancer detection 39.

Methods to isolate and detect EVs from urine can be very complex. At present, ultracentrifugation is the most convenient and economical method for EVs detection. However, ultracentrifugation can damage EVs and lead to relatively low yields and recovery rates 40. Differential centrifugation is the "gold standard" for purification of EVs, but it is time-consuming 41. Therefore, other methods, such as immunoaffinity 42 and microfluidic filtration systems 43, have been preferred as alternative methods or used in parallel with centrifugation methods.

EVs have huge potential in cancer detection applications. One of these cancer-related genes detected in urinary EVs is *SLC2A1*. Other genes, such as *GPRC5A* and *KRT17,* are overexpressed in stage pT1 and advanced BC 44. These results provide the potential for EVs as biomarkers for detecting non-muscle invasive bladder cancer and muscle-invasive bladder cancer. However, because the sample size is unevenly distributed and relatively small, the study still needs further evaluation of patients with pT2 and high-grade BC. EVs can also be used as a platform for diagnosing PC. By detecting EV-derived gene *TMPRSS2-ERG*, the overall diagnostic sensitivity of PC was 81%, and the specificity was 80%. Non-invasive detection of urinary EVs enables disease detection for patients without the need for diagnostic transrectal ultrasound 45.

Interestingly, miRNAs associated with EVs can also be biomarkers for diseases other than cancer. miRNAs from EVs can be used for the detection of acute myocardial infarction 46, 47, chronic hepatitis B 48, and their correlation with the presence of liver toxicity has been demonstrated in rat models 49. Despite the obvious advantages of using EVs for disease detection, the small sample size and difficulty of separation are still the limiting factors for widespread use 50, 51. Lack of existing technology for isolating EVs also hinder disease detection at an early stage.

## Current technologies for urine-based biomarkers

Urine biopsy has been termed as "liquid gold," as these samples are widely utilized as a rapid and non-invasive source for the molecular detection and surveillance of urological cancers. Routine urine analysis for disease diagnosis relies on reagent strips and microscopic examination of clinical urine samples. The presence or elevation of certain biomarkers, such as RBC, WBC, proteins, glucose, and ketones, may indicate the presence of pathological conditions 52. Histochemical tests cannot fully confirm the occurrence of the disease, so additional tests are needed to supplement these findings. Still, these examinations provide a rapid, simple, and relatively low-cost method for diagnosis and screening 53.

In the following sections, we focus on current and upcoming technologies available for biomarker detection through urine biopsy and discuss their advantages and disadvantages for clinical utility (**Table 2**).

### For physical biomarkers

#### Histochemical examinations

Urine cytology is non-invasive and remains a gold standard in the diagnosis of BC in clinical settings. However, the challenge with cytology is that it has low sensitivity for low-grade tumors (48-68%) 54 and is operator-dependent. There is a potential for false-positive results, which warrants further examinations. Therefore, cytology is often complemented by other tests in parallel. Counting urinary particles is another important part of a routine urinalysis, providing a non-invasive and inexpensive clinical significance 55. Urine microscopic examination is to identify and quantify the insoluble substances present in the urine under the microscope. Their presence at a high level may indicate a pathological status (**Table 1**).

Biochemistry examinations are not definitive tests. In the diagnosis of complex diseases and their symptoms, such as cancer, other screens are required to complement these findings. These tests include imageological examination and aspiration biopsy. The interpretation of results together with complementary tests are of great importance since many symptoms presented in cancer can be observed in other pathological events, such as inflammation. For example, an elevation of protein levels or the presence of protein crystals in the urine is detected via a urine dipstick assessment or urinalysis together with complete blood count (see **Section 3.41**). These results can determine the presence of urinary tract stones (nodular stones) or inflammatory diseases (such as leukocytosis). A pH change in urine may suggest the presence of other inflammatory conditions such as urinary tract bacterial infection or nephritis (inflammation of the kidneys) 56. The efficacy of histochemical examinations is also limited to advanced or metastatic cancers, as these events are not observed in the early stages of the disease. This limitation is usually due to the use of non-specific biomarkers in histochemical examinations, and the appearance of these biomarkers usually only occurs in advanced stages of the disease.

#### Size-based cell detection

Molecular analysis of biomarkers present in urine provides a powerful tool for disease diagnosis, but the isolation of biomarkers is challenging due to the highly heterogeneous composition of urine 57. Membrane-based filtration is one of the label-free methods for separating biomarkers based on their size and deformability. Membrane filters consist of many well-defined pores, where particles smaller than the pores can pass through them and particles larger than the pores are captured on the membrane 58. Some studies have reported the use of membrane filters to isolate urine biomarkers such as EV and BC cells 59, 60. However, whether at the micron or nanoscale, the filtration technology is often hindered by technical issues such as biofouling, low throughput, need of skilled handling and may damage rare cells in the process 61.

#### Antigen-based cell detection

Another example is the ImmunoCyt/uCyt+ assay. Exfoliated urothelial cells are one of the biomarkers for detecting BC. ImmunoCyt is an immunocytochemical assay that uses three types of fluorescent antibodies to identify antigens specific to EBCCs 62. One of these antibodies (19A211) targets the high molecular weight form of glycosylated carcinoembryonic antigens, while the other two antibodies (LDQ10 and M344) target glycosylated epitopes 63. These red and green fluorescences were read using a fluorescence microscope, and if red or green was observed in at least one cell, the sample was considered positive. Compared with traditional cytology tests (44.5% sensitivity and 96% specificity), ImmunoCyt test shows higher sensitivity (80%) but lower specificity (70%) 62.

### For protein-based biomarkers

An abnormal elevation of specific proteins in a patient's urine sample can be used in disease diagnosis. We discuss the types of single-protein marker assays or multi-protein biomarker assays commonly used in the following sections.

#### Lateral flow assays (LFA) for single antigens

LFA, also known as immunochromatographic tests, uses antibodies to immobilize flowing targets quickly, but are not as sensitive as conventional methods 64, 65. In the following sections, we discuss key applications of LFA using urine biopsy samples, namely the detection of BC and Streptococcus pneumonia (SP) infection.

##### Nuclear matrix protein 22 (NMP22)

NMP22 is overexpressed in BC cells and can be detected in EBCCs from urine. Bladderchek can aid in the diagnosis of bladder tumors by examining abnormal levels of NMP22 in the urine. It is a POC certified by the Food and Drug Administration (FDA). During the test, an untreated urine sample will be added to the device, and NMP22 in urine samples will react with colloidal gold-conjugated monoclonal reporter antibodies and form immune complexes. Monoclonal antibodies in the test zone will capture the resulting immune complexes. If the NMP22 protein content in the urine is high enough (concentrations above 10U / ml), a visible line will form. If the control line is present in the device, the result is considered positive 66.

The overall sensitivity and specificity for bladderchek were 56% (52-59%) and 88% (87-89%), respectively. However, the LFA demonstrates low sensitivity in detecting low-grade cancer. When comparing bladderchek with cytology, the sensitivity of bladderchek was slightly higher, while the specificity was slightly lower than cytology 66, 67. For example, in 1300 patients, the sensitivity and specificity of bladderchek were 55.7% and 85.7%, respectively, while the sensitivity and specificity of cytology were 15.8% and 99.2%. Although NMP22-based Bakerchek is inexpensive and portable, the low predictive accuracy due to false positives limits its use as the gold standard for BC diagnosis, especially in low-grade cancers. Thus, bladderchek can only be used as an additional measure in combination with other tests to detect BC.

##### Bladder Tumor Antigen (BTA)

BTA has been proven to be an effective biomarker for BC testing. BTA stat ™ and BTA TRAK™ are two commercial methods based on BTA that can detect an antigen called complement factor H-related protein in urine samples 68. The difference between the two assays is that BTA stat is a cartridge-form enzyme immunoassay that can qualitatively identify the target protein, while BTA TRAK is a sandwich-type immunoassay that exists in a 96-well format and can qualify the amount of target protein in urine samples.

BTA stat detects antigens in urine samples to form antigen-binding complexes, which can also be represented by visible lines in the test zone. In contrast, the presence of antigens and the reliability of results are demonstrated by control lines. After the captured monoclonal antibody specifically binds to the complement factor H-related protein and completes the enzymatic reaction between the alkaline phosphatase-reported monoclonal antibody and the binding complex, the absorbance needs to be measured. When the abnormal range is higher than 14 U/ml, the absorbance will indicate the concentration of the antigen 69, 70. A comparative study of BTA stat and BTA TRAK shows that when detecting BC, the overall sensitivity of BTA TRAK is slightly higher than that of BTA stat (77.5% to 65.3%). In comparison, the overall specificity of BTA TRAK is 62.5% and 71.8%, respectively 69. Although both methods show low sensitivity in detecting low-grade tumors or low-stage cancers, it should be emphasized that in this study, BTA TRAK performed better than BTA stat.

Due to the small sample size and the lack of significant results from current research, it is theoretically difficult to determine their overall efficacy in this application. Reports suggest that both BTA stat and BTA TRAK show better sensitivity in cytology 70, 71, especially in low-grade or low-stage tumors. Low specificity is the main disadvantage of BTA-based tests. Besides, as complement factor H protein is a serum factor, the presence of hematuria can lead to false-positive results 72. Similar to Bladdechek, both BTA stat and BTA TRAK cannot be regarded as a definitive screen for BC detection and must be used in combination with cystoscopy.

##### SP antigen

**Figure 1:**
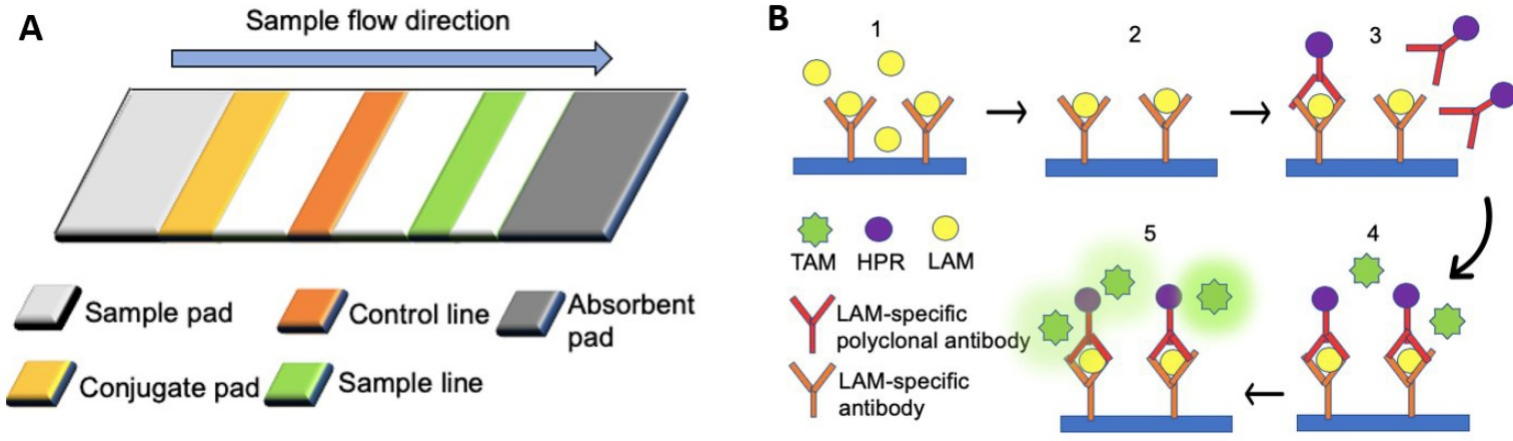
** Lateral flow assays for the detection of biomarkers associated with multiple disease types.** (A) The rabbit anti-SP antibodies (primary antibodies) of a lateral flow assay were immobilized on the sample line (orange), and the goat anti-rabbit antibodies (secondary antibodies) were immobilized on the control line (green). Rabbit anti-SP antibodies conjugated with colloidal gold particles were fixed by fibrous support (yellow), but they were mobilized when fluid was introduced from the sample pad 65. (B) An ELISA assay. (1) The urinary sample was added to the substrate coated with LAM-specific antibodies. (2) Mobilized particles were removed by washing with PBS (3) Horseradish peroxidase (HRP) -conjugated LAM-specific rabbit polyclonal antibodies were applied to the substrate, followed by the removal of all mobilized particles with PBS. (4) Tetramethylbenzidine (TMB) was added to the substrate. (5) The enzymatic reaction between TMB and HPR produced a colorimetric signal.

SP infection can lead to morbidity, with high mortality rates particularly observed in developing countries 73, 74. Early detection of SP through rapid pneumococcal urinary antigen tests is crucial to allow faster administration of antibiotic treatment 64. The BinaxNOW urine-based test for S. pneumoniae (BinaxNOW-SP) is a commercially available kit that can rapidly detect the presence of SP antigen, namely pneumococcal C-polysaccharide, present in the urine of SP-infected patients (**Figure 1A**) 64, 65. When SP (target) is present, the conjugated rabbit anti-SP will bind to SP, forming a conjugated complex, which flows to the sample line, allowing the immobilized primary antibodies to bind the conjugated complex. As the sample keeps flowing, the excess conjugated rabbit anti-SP antibodies will be captured by the secondary antibodies on the control line. A positive result will be marked by color changes on the sample and control lines due to the immobilized conjugated gold particles. In contrast, the negative result is indicated by color change only on the control line 65. Studies on LFA, such as the BinaxNOW-SP, have reported moderate sensitivity rates (74%) but high specificity rates (97.2%) 64. One of the key advantages of LFA is the time required, as the test can be completed within 15 min. Such tests are more rapid than conventional cultural methods that require at least 24 hours of incubation. Hence they are preferred over the conventional cultural methods, which are almost as specific (98.6%) but less sensitive (59.4%) than LFA 64.

#### Multiplex protein biomarker panels

LFAs for the detection of single antigens in urine biopsy lack the specificity of cytology. Still, multiplex protein biomarker panels can improve such drawbacks by combining abnormally elevated biomarkers in cancer patients. According to a study of 125 patients, ten biomarkers (IL8, MMP9, MMP10, SERPINA1, VEGFA, ANG, CA9, APOE, SERPINE1, and SDC1) were detected from urine samples on one multiplexed assay, and its overall sensitivity and specificity for BC detection are 79% and 88%, respectively 75. Other biomarkers such as Coronin-1A, apolipoprotein A4, semenogelin-2, gamma synuclein, and DJ-1 / Park7 were also overexpressed in patients with transitional bladder carcinoma. ELISA (sensitivity: 79.2%, specificity: 100%) and Western blot (93.9%, specificity: 96.7%) confirmed that the biomarker panel demonstrates high sensitivity and specificity even in low-grade (Ta/T1) BC, as compared to LFA assays 76.

### For genetic biomarkers

#### FISH

FISH can allow the detection of abnormal gene expression in cells based on the hybridization process between fluorescently labeled DNA probes and the DNA target sequence. Commercial kits to identity chromosomes *CEP3*, *CEP7*, *CEP17*, also the *9P21* (P16) LSI are available for research (UroVysion, Abbott Molecular, Inc., Des Plaines, IL). The FDA approved them in 2001 for clinical use with patient-derived EBCCs in the urine.

Studies demonstrated the high detection sensitivity of the UroVysion FISH assay (84.2%) in patients with urothelial cancers. This detection method was also highly specific (91.8%) in patients with cancers who have underlying genitourinary disorders 77. Compared with cystoscopy (67%), FISH analysis is also more sensitive in detecting BC (87%) 78. For all other types of urothelial carcinoma, according to the Meta-analysis study, the sensitivity of FISH was around 72% 79. Such high sensitivity and selectivity rates suggested that FISH could aid in the diagnosis of BC for patients presenting with equivocal cytology. However, recent studies have shown that a larger sample size is needed to derive the sensitivity and specificity of UroVysion 80, and more research should be performed to refine the results.

Furthermore, the presence of some of these chromosomal and morphologic changes was not clearly understood. For the 9p21 chromosome, a positive result is considered when ≥ 12 cells show no 9p21 signal 77. However, the presence of tetraploid in the FISH analysis may reflect normal cell division or proliferative response of the bladder epithelium to inflammatory damage rather than cancerous processes. These challenges hinder the clinical utility of FISH analysis as the ultimate tool for disease detection.

#### Oligo-conjugated magnetic particles (MPs)

The messenger RNA of the known prostate cancer antigen 3 (*PCA3*) gene is used as a biomarker for detecting PC in urine samples. Early diagnosis of PC is based on the detection of prostate-specific antigen (PSA) in serum, but the PSA test lacks specificity 81. Therefore, PCA3 dominates the detection of PC. Progensa PCA3 is a commercially available kit that has the potential to detect PC in a non-invasive manner 82.

The process is divided into three parts: target messenger DNA capture, transcription-mediated amplification (TMA ™), and amplification of target RNA by amplicon detection 83. Specific oligonucleotides are hybridized to the target in whole urine and captured on MPs. After magnetic separation and washing procedures are completed, the purified RNA is amplified by TMA. TMA uses target exons 3 and 4 as primers. Moloney murine leukemia virus reverse transcriptase and T7 RNA polymerase was used for amplification. The luminescence produced by the hybridization probe was quantified using a luminometer, and the results were obtained. Progensa PCA3 test has moderate sensitivity and specificity of 69% and 79% with urine samples, which is much higher than the PSA serum test (specificity: 28%) 83. Compared with standard gene amplification methods, the advantages of using oligonucleotide-conjugated MPs for detection include quantitative analysis and ease of handling, and the use of whole urine can reduce the need for pretreatment of urine samples 83.

#### PCR analysis

PCR-based methods can detect mutant DNA and RNA genes in urine samples to diagnose corresponding diseases, such as BC 84 and PC 85. Conventional PCR, qPCR, and hot-start PCR use DNA as templates for replication, while RT-PCR and RT-qPCR use RNA to transcribe complementary DNA (cDNA) for use as a template. When mRNA, long noncoding RNA, and small RNAs are involved, reverse transcription will be carried out in a similar way to transcribe cDNA using DNA-dependent DNA polymerase. PCR has been used for many different types of biomarkers detected in urine. For example, the detection of CD44 gene abnormality (biomarker reflecting cancer malignancy) through PCR-based technology can achieve high sensitivity of 91% and a specificity of 83% 86. PCR-based methods can also detect telomerase mutations in urine, such as the somatic telomerase reverse transcriptase (TERT) promoter mutations (biomarkers associated with cancer recurrence), with an overall sensitivity of 80.5% and specificity 89.8% 87. Currently, with the development of high-throughput sequencing methods, traditional PCR-based methods have lost their dominance. However, it is still a convenient and economical method compared to next-generation technology. In the recent decade, advances in the area of molecular techniques have led to the development of modified techniques, such as dPCR 88. In this process, target DNA molecules are diluted to a single-molecule level and distributed averagely in an individual compartment before PCR amplification. After amplification, dPCR can detect each compartment with a mutation sequence according to the fluorescent signal. It is more sensitive than qPCR because it can focus on single nuclei molecules. However, the precision of dPCR depends on the false-positive rate, the ratio between mutant and non-mutated sequence, and the analyzed compartment numbers 89.

Droplet digital polymerase chain reaction (ddPCR) is an improved form of PCR technology based on the principle of dPCR. It can also be used to detect cancer of the urinary system 90. In ddPCR, the targets DNAs are compartmentalized within independent water droplets due to the surface tension and shear force between the oil and aqueous phase. The countless aqueous droplets (~20,000) are collected for amplification. Subsequently, fluorescent signals from each droplet will be detected 91. ddPCR can accurately determine the nuclei acids components and can achieve sensitivity to the point of detecting one allele frequency detection from 100,000 molecules 92.

BEAMing is another modified dPCR based method and can detect low cfDNA mutations of up to 0.01-0.02% 93. During the process of BEAMing, biotinylated oligos are first bound to streptavidin-coated magnetic beads. The primer-bound beads are then mixed with template DNA and an oil and detergent mix to create microemulsions. The process of separating single DNA works similarly as compared to ddPCR. Following which, DNA amplification will commerce, and the bead-bound oligonucleotides serve as primers during the reaction. Once the beads are released from the emulsion and purified using a magnet, the fluorescently labeled antibodies will label the hybridization probes for flow cytometry detection 94.

#### NGS

NGS is a high-throughput method for the detection of DNA and RNA mutations. As compared to Sanger sequencing, NGS can detect multiple samples and simultaneously provide large amounts of genetic variation information. This facilitates the parallel detection and analysis of multiple genes. In studies involving the detection of fibroblast growth factor receptor 3 (FGFR3) mutations from urine samples of cancer patients, NGS was proven to be much more sensitive than PCR-based methods for detection 95.

The first NGS platform was commercially available in the year 2005 (454 GenomeSequencer FLX instrument, Roche Applied Science) 96. The operating principle of GenomeSequencer is based on pyrophosphate detection. Recent technological advances improved the accuracy of detection (99.997%) by increasing throughput to 700 Mb with a higher read length of around 700 base pairs (bp). At present, the company "Illumina" has also developed sequencing platforms for RNA sequencing (RNA-seq).

There are two other typical approaches for NGS platform, one is based on sequencing by ligation, and the other is based on ion semiconductor sequencing. ABL SOLiD system invented by Applied Biosystems is based on the former method. Because different fluorescent colors label each base, the error is reduced during the sequencing process. The life technologies newly developed ligand-based 5500xl W System can even detect low-frequency variants in disease research with accuracy up to 99.99% and can conduct reliable RNA-seq.

However, NGS has been limited in terms of usage in a larger population, as the reagents used in the pyrosequencing process can be quite costly. Other limitations of NGS are mainly focused on the interference background signal, error rate of amplification, and the potential incomplete chemical reaction during amplification.

UroSEEK is an example of next-generation sequencing for urine samples. UroSEEK is a massively parallel sequencing-based assay consisting of three components 97. The first part of UroSEEK is called UroSeqS, which can detect ten mutant genes (*FGFR3*, *TP53*, *CDKN2A*, *ERBB2*, *HRAS*, *KRAS*, *PIK3CA*, *MET*, *VHL*, and *MLL*) in urothelial tumors. On the other hand, TERTSeqS can detect urethral epithelial cell activation mutant *TERT*, while FatSeqS can detect aneuploidy. UroSEEKs' overall sensitivity is 83%, which is still much higher than standard cytology for early BC detection. UroSEEK is also very specific (93%), highlighting its potential in early BC detection. Similarly, in the diagnosis of low-grade tumors, UroSEEK detected 67% of low-grade tumors. Still, none of them were detected by cytology, indicating that UroSEEK dominates early BC and low-stage BC detection. The specificity obtained when using UroSEEK to test 188 healthy individuals was 99.5%. UroSEEK has a moderate sensitivity of 75% when applied to the surgically treated population, which means UroSEEK has potential in relapse BC detection 98.

Another example is Capp-Seq, which is a high-throughput sequencing method for BC detection and monitoring, using CfDNA from urine samples. cfDNA can be extracted by a Q resin-based method 99, 100, followed by mixing with the Q-sepharose resin slurry and discarded after centrifugation. In the early BC test cohort (<pT2), the sensitivity of the CAPP-Seq test mutations (*TERT* and *PLEKHS1*) was 83%, which was much higher than the results of cytology (14% sensitivity). CAPP-Seq also achieved a high specificity of 97% (33/34). The sensitivity to the pTa result was 77.5%. The effectiveness of CAPP-Seq in monitoring BC recurrence was also tested. In the mutation detection cohort, CAPP-Seq achieved 84% high sensitivity and 96% specificity. If mutations in the tissue were detected in the cohort in advance, the sensitivity was 91%, and the specificity was 100%, indicating that CAPP-Seq is highly accurate when used to detect recurrence of BC.

### For chemical-based biomarkers

#### Dipstick Urinalysis

For chemical-based biomarkers, dipstick urinalysis provides a simple, rapid, and inexpensive approach for screening. Still, since false positives and false negatives may occur other tests are required to confirm the results 53, 101. Dipstick urinalysis has been widely available commercially for clinical and POC applications 102. The working component involves an absorbent pad infused with chemicals that will react with specific urinary constituents such as ketone, protein, and glucose. The reactions lead to color changes that can signify abnormalities in the levels of these constituents 101. For example, the presence of bilirubin or nitrite in the urine produces a pink (azo dye) in the test strip, indicating liver disease or bacterial infection 53, 103. Ketones in the urine can form a purple color on the test strip, which may suggest the presence of type 1 diabetes 53. Similarly, the blue color formed in the test strip may indicate renal insufficiency due to the detection of albumin 101. The time required to observe the colorimetric intensity will be compared with the color code table specified by the manufacturer.

### For lipid biomarkers

#### ELISA

Tuberculosis (TB) is the leading cause of death worldwide, with 10 million new cases reported each year 104. Chest radiology and sputum smear microscopy are the most widely used diagnostic methods for cases in developing countries, accounting for more than 90% of all TB cases worldwide. However, these diagnostic techniques are often unreliable or unavailable in areas with limited resources, so there is a need to develop a rapid, easy-to-use, and biomarker-based detection method 105.

Lipoarabinomannan (LAM) is the only TB biomarker listed by WHO and can be detected in the urine of TB-infected patients. To detect the presence of LAM in the urine, "Clearview TB ELISA" is the commercially available protocol based on the sandwich ELISA (**Figure 1B**) 106. The presence of LAM in the sample will yield immobilized horseradish peroxidase (HRP) conjugated LAM-specific rabbit polyclonal antibodies, thereby generating a colorimetric signal. The presence of other underlying diseases can affect the sensitivity of the assay. ELISA can achieve high specificity (97%), albeit the overall sensitivity is low (14%). In patients co-infected with TB and human immunodeficiency virus (HIV), the specificity and sensitivity of the test become different due to elevated levels of LAM found in the urine of immunosuppressed patients. Among HIV-positive patients, the sensitivity of ELISA is higher (51%), but the specificity is lower (94%) 105.

## Upcoming technological advancements to facilitate biomarker detection in urine

Traditional approaches to isolate biomarkers for analysis include precipitation, filtration, and centrifugation, and these methods are often subjected to inherent drawbacks such as membrane clogging, low throughput, and lack of automation 61. Precipitation is one of the most common approaches for particle isolation and is based on the solubility of various substances in different solvents. Generally, the substance with lower solubility will be precipitated 107. The resultant solid-liquid fractions can then be separated by physical methods such as filtration or centrifugation 108. Filtration is the use of membrane with well-defined pores (membrane filter) or arrays with well-defined gap sizes (micro-array filter). Particles larger than the pore size are retained 109. Centrifugation is the application of centrifugal force generated by a rotor to separate substances with different densities, and particles with higher densities will sink relatively faster.

With the advancement of technology, new methods are now actively screened to allow better retrieval of biomarkers to enhance the sensitivity and specificity of disease detection.

In the following sections, we detail the incorporation of technological trends, such as microfluidics and affinity binding assays that could facilitate the enrichment of biomarkers for detection.

### Microfluidics

Microfluidics is the manipulation of small amounts of fluids in microscale channels. It can be classified as an active system or a passive system. Examples of active systems include acoustophoresis, magnetophoresis, and dielectrophoresis, while examples of passive systems include fluid dynamics, gravity separation, and inertial microfluidics. Briefly, acoustophoresis is a label-free method which applies ultrasonic fields on the microchannel where particles are focused either on the pressure node or the anti-node, due to the size dependence of acoustic forces 110. Contrary to acoustophoresis, magnetophoresis is a label-based approach of which specific targets labelled by antibody-coated magnetic beads will be immobilized or concentrated on designed outlets under the external magnetic field to achieve the isolation of specific labelled targets 111. Dielectrophoresis is the use of non-uniform electric fields, such that polarizable particles with permanent or induced dipole within a microchannel will experience a net force, leading to particle separation 109. Systems based on the principles of gravitational separation allow particles with different sizes to be separated as they experienced different accelerations rates perpendicular to the direction of the microchannel flow 112.

Due to the benefits of a microscale dimension, such as a larger surface-area-to-volume ratio, many physical phenomena such as laminar flow, Dean flow, inertial focusing, and rapid diffusion, are more apparent. They can be used for a wide variety of applications 113. These advancements revealed new opportunities in the area of POC applications as well as improved the feasibility of disease monitoring due to the relatively low fabrication cost.

#### Inertial microfluidics

When Reynolds number is in the range of 1-100, the inertial effect becomes significant 109. The inertial lift effect is the balance between shear-gradient and wall-induced forces, allowing particles to focus within a narrow path in a straight channel. If a curvilinear channel is used, secondary inertial forces such as counter-rotating vortices are formed by centrifugal forces 114. Because inertial focusing effects depend on size, particles of different sizes flowing through the channels would be focused at different positions 61.

Recent studies have employed inertial microfluidics to specifically separate EBCCs from bladder wash urine 115. Such techniques were previously optimized for the detection of circulating tumor cells (CTCs) from blood-based liquid biopsy 116. After a pre-processing step to remove larger impurities such as squamous epithelial cells (30-60 μm) via filtration, the microfluidic device concentrated and processed the samples at a rapid rate of 1.7 ml/min to separate the target EBCCs (11-15 μm). The device could be multiplexed to potentially complete the enrichment of a 50 ml sample under 10 min. The device demonstrated high sensitivity (93.3 ± 4.8%), and the cells collected remained viable.

#### Three dimensional (3D) traps for capture

Complex 3D microfluidic chips have been designed for the detection of cancer cells and exosomes. In recent studies, the integration of functional trace elements is achieved through colloidal self-assembly, which is called multi-scale integration through design self-assembly 117. As the structure of the chip provided a large surface area for interaction, near-surface hydrodynamic resistance was decreased 118, 119. Studies with spiked standards suggested that the nano-HB chip demonstrated a detection limit of 10 exosomes per μl. Subsequent clinical screens were able to detect markers for ovarian cancer, such as circulating exosomal CD24 (100 fg ml^-1^), EpCAM (10 fg ml^-1^), and FRα (10 fg ml^-1^), using only a small amount of plasma (2 μl). Studies specifically on the use of urine-based biopsy have also been reported, although more studies were focused on the detection of common analytes such as glucose, dopamine, and epinephrine 120. Although such microfluidic technologies can achieve low detection limits, the fabrication of patterns is complicated and expensive 117, 121.

#### Deterministic lateral displacement (DLD) devices

**Figure 2:**
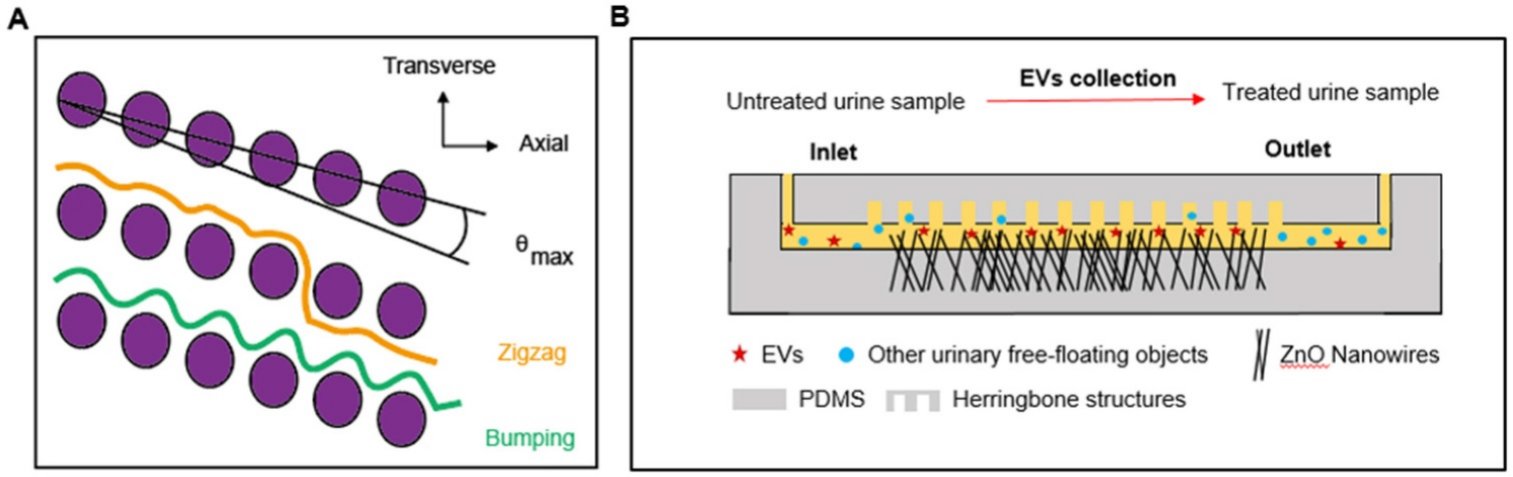
** Microfluidics to facilitate the detection of biomarkers from a urine-based liquid biopsy.** (A) In a DLD device, particles move either in a transverse mode (orange) or bumping mode (green), as shown. The diameter of particles and nominal critical diameter is characterized as D_P_ and D_C,_ respectively. When D_P_ < D_C_, particles follow the laminar flow in a zigzag mode (θ = 0, orange), while for D_P_ ≥ D_C_, particles follow a bumping mode (θ = θ_max_, green). (B) In the nanowire-anchored microfluidic device, untreated urine samples are injected into the inlet of the device, the EVs in urine samples were captured by anchored ZnO nanowires based on electrostatic interactions. At the same time, uncollected urinary free-floating objects are collected in the outlet 126.

Microfluidic devices based on the principles of DLD have been developed for the separation of CTCs, blood cells, parasites, and bacteria 122-124. In a recent study, to expand the range of applications, the technology was manufactured at the nanoscale and used to sort exosomes. In this technique, parameters such as the row-to-row shift, δ, pillar pitch, λ, pillar gap size, post-gap (G), and maximum displacement angle, θ_max_ (0<θ<θ_max_) determined the flow of particles downstream (**Figure 2A**). Displaced particles moved towards the collection wall along the length of the array. For a given G, ranging from 25 nm to 235 nm, particles with various D_P_ performed different θ, allowing the sorting of particles with sizes from 20 nm to 110 nm. Comparable displacement distribution was displayed with the detection of exosomes from 10 μL human urine 123. Despite the sensitivity of detection, DLD has not been widely used in clinical applications, due to the intricate fabrication patterns required.

#### Nanowire-based microfluidics

Nanowire electrodes or sensor arrays have been designed for the detection of biomarkers from serum for cancer detection 125. Recent studies suggested that ZnO nanowire-based methodology can be used to collect urinary EVs, with an estimated collection efficiency of 99% (**Figure 2B**) 126 and miRNA encapsulated in EVs will be extracted for further analysis. Urine was injected into the device through the inlet, and the anchored nanowires collected EVs from the urine sample based on electrostatic interactions between positively charged nanowires and negatively charged urinary EVs. Detection efficiency was > 99%, demonstrating superior sensitivity as compared to standards of EV isolation, such as centrifugation.

#### Integrated POC systems

A microfluidic system that combines the isolation, characterization, and quantification steps into a single platform is essential to POC applications 127. In recent studies, an integrated microfluidic device was used to carry out isolation, enrichment and quantification of urinary cells and EVs from BC patients simultaneously, through a double-filtration approach (pore size of 200 nm and 30 nm) 43. The captured urinary EVs were analysed *in situ* by on-chip ELISA. The colours generated from the ELISA assay could be captured by an application on the phone, providing a relative quantification of EV concentration based on colour intensity. The double-filtration device reported a high sensitivity of 81.3% and specificity of 90%, based on a cohort of BC patients and healthy controls. Relatively large sample volumes (8 ml) could be handled under continuous flow, subject to the potential biofouling of membrane pores. Another study combined microfiltration and microchip enzyme-linked immunosorbent assay (ELISA) methods to collect EBCCs for BC detection. The device reported a sensitivity of 77.1% and a specificity of 90% 128. Overall, even though some of these microfluidic-based techniques can achieve high separation efficiency, certain parameters such as the use of external fields may lead to low throughput. For example, an increase in flow rate will decrease the time for the biosensor to recognize its targets 113. The fabrication of some microfluidic devices may also require higher costs due to the complex geometries required 61, 129.

### Fluorescence-based Analysis

Fluorescence detection is one of the most powerful approaches for biochemical detection. For example, it has been integrated with Western blot and ELISA to detect specific binding between antibody and receptors 130. However, for the detection of complex samples such as urine, other components will also emit fluorescence when the fluorescent label is excited, resulting in high background noise. Recently, long persistent phosphors (LPPs) had been successfully applied to fluorescent biosensors to achieve a higher signal-to-noise ratio. As compared to conventional phosphors, long persistent (or afterglow) phosphors can emit visible, near-infrared, or UV light for a much longer duration after the cessation of the light excitation 131. Here, we discuss some of the successful examples of utilizing LPPs for biomarker detection in the following section.

#### LPPs combined with photonic crystal substrate

**Figure 3:**
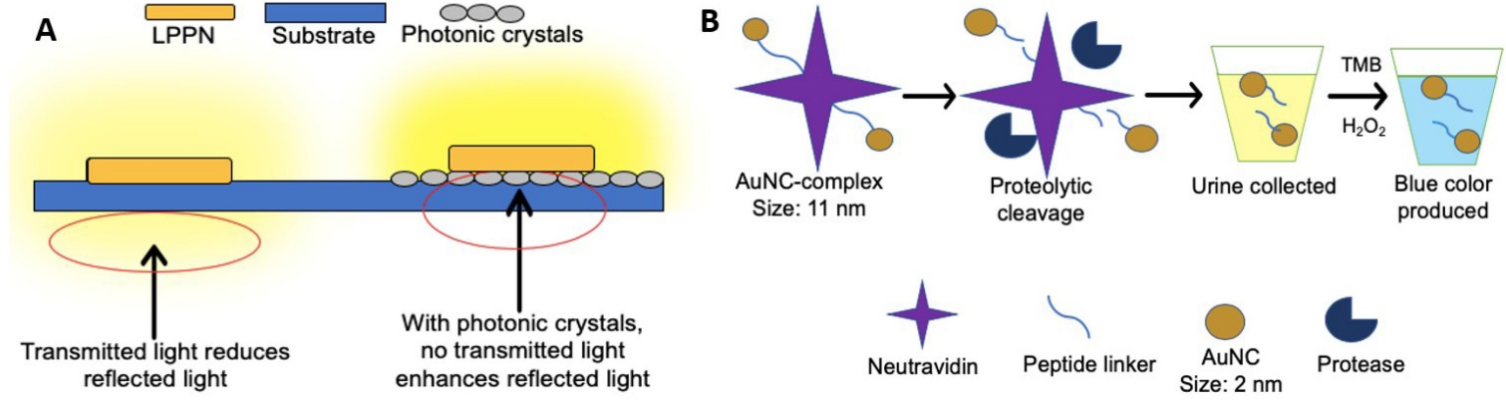
** Assays based on the fluorescence-based analysis.** (A) In an optical sensor using long persistent phosphors (LPPs) and photonic crystals as a substrate, signal enhancement via photonic crystals substrate minimizes transmitted light, resulting in the enhancement of reflected luminescent signal. The light emitted by the LPPN penetrates the substrate (blue) without photonic crystals but cannot penetrate the substrate in the presence of photonic crystals (grey). (B) In a procedure based on the use of renal-clearable catalytic gold nanoclusters, tumor-associated proteases cleave the peptide linker between the gold nanoparticle complex and neutral avidin. The presence of AuNCs is tested by adding hydrogen peroxide and 3,3',5,5'-tetramethylbenzidine (TMB) to the urine as AuNCs catalyse the reaction between hydrogen peroxide and TMB. The blue colour produced indicates the presence of AuNCs found in urine (cancer presence).

An optical sensor using photonic crystals as a substrate was developed to detect BC-related miRNA-21 in urine 132. Briefly, the long persistent phosphors' nanoparticles (LPPNs) were added to single-stranded DNAs (cDNAs) that are fully complementary to miRNA-21, followed by the partial hybridization with back-hole-quencher-label DNAs (BHQ-DNAs). The fluorescence was quenched due to the FRET between LPPNs and BHQ dyes. When the miRNA-21 (target) was presented, BHQ-DNAs would be detached from cDNAs because of the higher binding affinity of miRNA-21 compared to BHQ-DNAs. Therefore, the fluorescence of LPPNs was restored as the removal of BHQ-DNAs. After the excitation is stopped, the fluorescence of LPPNs can be retained, and the autofluorescence emitted by other biomolecules will quickly decay, thereby reducing the background noise. Besides, since the photonic crystal substrate can reflect light with a peak wavelength near LPPN 535 nm, the light emitted by LPPNs will not be transmitted to the photonic crystal substrate, so the emitted light is maximized, thereby improving detection sensitivity (**Figure 3A**). In this study, a detection limit of 26.3 fM was reported, and at least 97.33% of the known miRNA-21 concentration can be recovered.

#### LPP semiconducting nano-polycomplex sensor

In addition to miRNA, LPP is also successfully applied for urinary exosome detection to achieve low background noise. A research team has developed an afterglow semiconductor polymer nanocomposite (ASPNC), which is synthesized by electrostatic attraction between afterglow semiconductor polymer nanoparticles (LPP probes) and aptamers labelled with quenchers 130. The fluorescent signal of ASPNC was quenched because of the close distance between aptamers' quenchers and the nanocomplexes. Due to the high binding affinity of knockout aptamers, they will interact with specific targets on exosomes (CD36, EpCAM, HER2, and MUC1), thereby increasing quenching in the presence of targeted exosomes (HeLa exosomes). The fluorescent signal would be turned on due to the increase in the distance between aptamers' quenchers and nanoparticles. The fluorescent signal remained due to its afterglow nature, and thus the signal could be detected after excitation, thereby minimizing the background noise generated by impurities. In the detection of exosomes in cell cultural medium, their experiment demonstrated the decrease in the limit of detection (LOD) nearly two orders of magnitudes compared to conventional fluorescent detections previously. Although the aptamer sequences of ASPNC can be easily altered to achieve high flexibility for different targets, the selection of accurate sequences is challenging 130, 132.

#### Renal clearable catalytic gold nanocluster

The biodistribution of proteins within the human body depends on the hydrodynamic diameters of proteins. For renal filtration, the diameter of proteins smaller than 5 nm will be excreted by urine rapidly, while the proteins with larger than 15 nm will be prevented by renal filtration 133. By capitalizing on these differences, a simplistic but innovative technology for cancer detection with urine-based liquid biopsy was designed 134. In this study, the team designed a complex made of neutravidin peptide-linked with ultra-small gold nanoclusters (AuNCs). The peptide linker could be cut by a protease named matrix metalloproteinases (MMPs), which is a biomarker released by some types of tumors such as colon cancer (**Figure 3B**). In the presence of MMP, AuNCs less than 5 nm will be released from the urine within one hour. However, in the absence of tumors, large AuNCs complexes (approximately 11 nm) cannot be transferred to the kidney system within an hour. As a proof of concept, the complexes were injected into mice with and without tumors, respectively. Within an hour, the blue color in urine produced by cancer-bearing mice could be observed, while no color change was observed in urine produced by healthy mice. Similarly, the complex and AuNCs not found within four weeks after injection indicate that the body has cleared it. Hence there were no reports of toxicity. Although this study demonstrated great potential for cancer detection, more research is needed due to the involvement of complicated physiological conditions in humans 134.

## Discussion

### Future involvement of microfluidics for disease detection

Cell separation methods based on conventional antibodies can achieve high specificity and sensitivity. Nonetheless, they are still limited by the high cost of antibodies and the possible damage to the target during the labeling process 114, 135. Due to these factors, many label-free microfluidic methods have been developed in the past decade, such as pinched flow fractionation, DLD, hydrodynamic filtration, and inertial microfluidics 109, 114. Among them, inertial microfluidics is one of the most promising techniques for cell-based detection, and several applications in disease detection have already been demonstrated 136-138. However, for the detection of nanoscale biomarkers such as EVs, RNA, and DNA, other principles such as nanofabricated microfluidic devices or fluorescence-based analysis may be more applicable 139.

### The role of artificial intelligence in POC applications

Clinical outcomes obtained by observations are subject to personal perception, which may lead to biased or inaccurate assessments 140. In the modern age, many people are equipped with portable digital technology such as smartphones that come high-resolution cameras, processors, and scanners. These components have been used for POC urine diagnosis to avoid colorimetric misinterpretation, thereby increasing sensitivity and specificity 140, 141. However, the lack of colorimetric consistency between different mobile phone models remains a key challenge to be solved 142, 143. Using external accessories of smartphones for detection to unify standards may be one of the solutions to this problem 143. However, at this point, a limited number of phone models can support these applications, and the external accessories also come with additional costs 144. Despite these limitations, artificial intelligence through portable digital technology is expected to play an important role in the future development of POC tests for urine-based liquid biopsies.

## Figures and Tables

**Table 1:** List of common urinary biomarkers

Type	Biomarker	Disease(s)	Advantages	Disadvantages	Refs.
Cells	RBCs	Varied, e.g., Glomerular membrane damage	Defined morphology; Complements downstream analysis	High phenotypic heterogeneity; Low and varied abundance	52
	Neutrophils	Varied, e.g., Prostatitis, urethritis			101
	Other WBCs, e.g., monocytes, histiocytes				
	Clue cells	Infection by the bacterium			101
	Transitional epithelial cells	Malignancy or viral infection			101
	Renal tubular epithelial cells	Necrosis of renal tubules			101
cfDNA	Varied (e.g., EGFR, KRAS)	Varied (e.g., myocardial infarction)	Ease of access; Complementary to molecular characterization	Short half-life; Low abundance; Low specificity	145-149
Exosome	NA	Varied, e.g., peripartum cardiomyopathy	Stability; Large range of biomarkers	No specific method of detection; Size overlap with other EVs	150-155
Others, e.g., minerals	Urine crystals	Varied, e.g., Inherited cystinuria	Low cost and rapid	Sensitive to patient dietary habits	101
	Urine casts	Varied, e.g., Chronic renal disease			101

Abbreviations: RBCs = Red blood cells; WBCs = White blood cells. NA = Not applicable; cfDNA = cell free DNA; EV = extracellular vesicles.

**Table 2:** Current and upcoming technologies for the analysis of liquid biopsies

Technology	Sensitivity	Specificity	Advantage	Disadvantage	Ref
**Physical - based**					
Filtration	EBCC: 87%	ND	Label-free, ease of operations	Low throughout, possible damage of cells	58
Antigen-based cell capture	EBCC: 80-100%	EBCC: 70-99%	Targeted capture	High cost, loss of target cells with low antigen levels	18, 62
**Protein-based**					
LFA	SP: 74%; NMP22: 52-59%	SP: 97.2%; NMP22: 87-89%	Rapid (< 15 min), ease of operations, POC	Require precise antibody preparation, sensitivity limited by sample volume	64, 66
**Genetics-based**					
FISH	72-87%	91.8%	Ease of operations, validated procedures	Operator-dependent	77-79
PCR	FGFR: 11.6% *CD44*: 91%	*CD44*: 83%	Economical, ease of operations, validated procedures	Limited dynamic range, Moderate rate of false positives	86, 95
NGS	FGFR: 60%; UroSEEK: 75-83%; CAPP-Seq: 77.5-91%	UroSEEK: 93-99.5%; CAPP-Seq: 96-100%	High throughput, Parallel detection	High cost, Digital background interference	99, 100
**Chemical-based**					
Dipstick Urinalysis	ND	ND	Low cost, rapid, ease of operations, POC	Confirmatory test required, potential false outcomes	53, 101
Cell-based					
Sandwich ELISA (protein or lipid)	LAM: 14-51%	LAM: 94-97%	Does not require purification of sample	Requires antibody optimization due to cross reaction	105
**Microfluidic assays**					
Inertial focusing	93.3 ± 4.8%	ND	Label-free, high throughput	Require sample concentration	115
3D traps	ND	ND	Low LOD: 10 exosomes per μl	Complicated fabrication of nanopatterns, time-consuming	117
DLD	ND	ND	Low LOD: 10 exosomes per μl	Intricate fabrication patterns	123
Integrated devices	BC: 81.3%	BC: 90%	POC	Cost, complex handling	43
**Fluorescence-based assays**					
LPPs-based sensor	miRNA-21: > 97.33%	ND	High signal-to-noise ratio, low LOD	Challenge in selecting suitable aptamer sequence	132
Catalytic gold nanocluster	ND	ND	POC, ease of operations, rapid	Not tested in humans	133, 134

Abbreviations: 3D = Three dimensional; DLD = Deterministic lateral displacement; LPP = Long persistent phosphors; LOD = Limit of detection; POC = Point of care; SP = Streptococcus pneumoniae; LAM = Lipoarabinomannan; LFA = Lateral Flow Assays. PCR = Polymerase chain reaction; NGS = Next-generation sequencing; FISH = Fluorescence in-situ hybridization; BC = Bladder cancer; SP = Streptococcus pneumoniae; ND = not determined; EBCC = exfoliated bladder cancer cells.

**Table A:** List of Abbreviations

EBCCs	Exfoliated bladder cancer cells
cfDNA	Cell-free DNA
EVs	Extracellular vesicles
WBC	White blood cells
RBC	Red blood cells
CC	Colorectal cancer
BC	Bladder cancer
PC	Prostate cancer
FISH	Fluorescence in-situ hybridization
ELISA	Enzyme-linked immunosorbent assay
PCR	Polymerase chain reaction
qPCR	Real-time polymerase chain reaction
dPCR	Digital polymerase chain reaction
NGS	Next gene sequencing
FGFR3	Fibroblast growth factor receptor 3
NSCLC	Non-small cell lung cancer
EBV	Epstein-Barr virus
miRNA	MicroRNA
LFA	Lateral flow assays
SP	Streptococcus pneumonia
NMP22	Nuclear matrix protein 22
POC	Point-of-care
FDA	Food and Drug Administration
BTA	Bladder Tumor Antigen
BinaxNOW-SP	BinaxNOW urine-based test for S. pneumoniae
MPs	Magnetic particles
PCA3	Prostate cancer antigen 3
PSA	Prostate-specific antigen
TERT	Telomerase reverse transcriptase
cDNA	Complementary DNA
ddPCR	Droplet digital polymerase chain reaction
RNA-seq	RNA sequencing
TB	Tuberculosis
LAM	Lipoarabinomannan
HIV	Human immunodeficiency virus
CTCs	Circulating tumor cells
3D	Three dimensional
DLD	Deterministic lateral displacement
LPPs	Long persistent phosphors
LPPNs	Long persistent phosphors' nanoparticles
cDNAs	Single-stranded DNAs
BHQ-DNAs	Back-hole-quencher-label DNAs
ASPNC	Afterglow semiconductor polymer nanocomposite
AuNCs	Gold nanoclusters
MMPs	Matrix metalloproteinases
LOD	Limit of detection

## References

[1] Murtaza M, Dawson SJ, Tsui DW, Gale D, Forshew T, Piskorz AM (2013). Non-invasive analysis of acquired resistance to cancer therapy by sequencing of plasma DNA. Nature.

[2] Chen L, Bode AM, Dong Z (2017). Circulating tumor cells: Moving biological insights into detection. Theranostics.

[3] Normanno N CA, Ciardiello F, De Luca A, Pinto C (2018). The liquid biopsy in the management of colorectal cancer patients: Current applications and future scenarios. Cancer Treat Rev.

[4] He M ZY (2016). Microfluidic Exosome Analysis toward Liquid Biopsy for Cancer. J Lab Autom.

[5] Herrmann N, Neubauer P, Birkholz M (2019). Spiral microfluidic devices for cell separation and sorting in bioprocesses. Biomicrofluidics.

[6] Md Ali MA, Ahmad Kayani AA, Majlis BY (2017). Biological Particle Control and Separation using Active Forces in Microfluidic Environments. Microfluid Nanofluidics.

[7] Botezatu I, Serdyuk Og, Potapova G, Shelepov V, Alechina R, Molyaka Y (2000). Genetic analysis of DNA excreted in urine: a new approach for detecting specific genomic DNA sequences from cells dying in an organism. Clin Chem.

[8] Bryzgunova OE, Skvortsova TE, Kolesnikova EV, Starikov AV, Rykova EY, Vlassov VV (2006). Isolation and comparative study of cell-free nucleic acids from human urine. Ann N Y Acad Sci.

[9] Togneri FS, Ward DG, Foster JM, Devall AJ, Wojtowicz P, Alyas S (2016). Genomic complexity of urothelial bladder cancer revealed in urinary cfDNA. Eur J Hum Genet.

[10] Mischak H DC, Vlahou A, Vanholder R (2015). Proteomic biomarkers in kidney disease: issues in development and implementation. Nat Rev Nephrol.

[11] Rzhevskiy AS, Razavi Bazaz S, Ding L, Kapitannikova A, Sayyadi N, Campbell D (2019). Rapid and Label-Free Isolation of Tumour Cells from the Urine of Patients with Localised Prostate Cancer Using Inertial Microfluidics. Cancers (Basel).

[12] Laguna TA WB, Starcher B, Luckey Tarro HK, Mann SA, Sagel SD, Accurso FJ (2012). Urinary desmosine: a biomarker of structural lung injury during CF pulmonary exacerbation. Pediatr Pulmonol.

[13] Sara Ongay MS, Peter Horvatovich, Jos Hermans, Bruce E (2016). Miller, Nick H.T. ten Hacken, Rainer Bischoff. Free Urinary Desmosine and Isodesmosine as COPD Biomarkers: The Relevance of Confounding Factors. Chronic Obstr Pulm Dis.

[14] Satyal U, Srivastava A, Abbosh PH (2019). Urine Biopsy—Liquid Gold for Molecular Detection and Surveillance of Bladder Cancer. Front Oncol.

[15] Andersson E, Steven K, Guldberg P (2014). Size-based enrichment of exfoliated tumor cells in urine increases the sensitivity for DNA-based detection of bladder cancer. PloS one.

[16] Bonberg N, Pesch B, Behrens T, Johnen G, Taeger D, Gawrych K (2014). Chromosomal alterations in exfoliated urothelial cells from bladder cancer cases and healthy men: a prospective screening study. BMC cancer.

[17] Croft W, Nelson C-E (1979). Collection and evaluation of normal exfoliated urinary bladder cells in man using scanning electron microscopy. Scand J Urol.

[18] Macgregor-Ramiasa M, McNicholas K, Ostrikov K, Li J, Michael M, Gleadle JM (2017). A platform for selective immuno-capture of cancer cells from urine. Biosens Bioelectron.

[19] Choi JJ, Reich III CF, Pisetsky DS (2005). The role of macrophages in the in vitro generation of extracellular DNA from apoptotic and necrotic cells. Immunology.

[20] Phallen J, Sausen M, Adleff V, Leal A, Hruban C, White J (2017). Direct detection of early-stage cancers using circulating tumor DNA. Sci Transl Med.

[21] Hirotsu Y, Yokoyama H, Amemiya K, Hagimoto T, Daimon H, Hosaka K (2019). Genomic profile of urine has high diagnostic sensitivity compared to cytology in non-invasive urothelial bladder cancer. Cancer Sci.

[22] Jain S, Lin SY, Song W, Su Y-H (2019). Urine-based liquid biopsy for non-urological cancers. Genet Test Mol Biomarkers.

[23] Reckamp KL, Melnikova VO, Karlovich C, Sequist LV, Camidge DR, Wakelee H (2016). A highly sensitive and quantitative test platform for detection of NSCLC EGFR mutations in urine and plasma. J Thorac Oncol.

[24] Song BP, Jain S, Lin SY, Chen Q, Block TM, Song W (2012). Detection of hypermethylated vimentin in urine of patients with colorectal cancer. J Mol Diagn.

[25] Su Y-H, Wang M, Aiamkitsumrit B, Brenner DE, Block TM (2005). Detection of a K-ras mutation in urine of patients with colorectal cancer. Cancer Biomark.

[26] Landau Z, Gross R, Sanilevich A, Friedmann A, Mitrani-Rosenbaum S (1994). Presence of infective Epstein-Barr virus in the urine of patients with infectious mononucleosis. J Med Virol.

[27] Chan KA, Leung SF, Yeung SW, Chan AT, Lo YD (2008). Quantitative analysis of the transrenal excretion of circulating EBV DNA in nasopharyngeal carcinoma patients. Clin Cancer Res.

[28] Arraud N, Linares R, Tan S, Gounou C, Pasquet JM, Mornet S (2014). Extracellular vesicles from blood plasma: determination of their morphology, size, phenotype and concentration. J Thromb Haemost.

[29] Pisitkun T, Shen R-F, Knepper MA (2004). Identification and proteomic profiling of exosomes in human urine. Proc Natl Acad Sci U S A.

[30] Théry C, Amigorena S, Raposo G, Clayton A (2006). Isolation and characterization of exosomes from cell culture supernatants and biological fluids. Curr Protoc Cell Biol.

[31] Doyle LM, Wang MZ (2019). Overview of extracellular vesicles, their origin, composition, purpose, and methods for exosome isolation and analysis. Cells.

[32] Crescitelli R, Lässer C, Szabó TG, Kittel A, Eldh M, Dianzani I (2013). Distinct RNA profiles in subpopulations of extracellular vesicles: apoptotic bodies, microvesicles and exosomes. J Extracell Vesicles.

[33] Caby M-P, Lankar D, Vincendeau-Scherrer C, Raposo G, Bonnerot C (2005). Exosomal-like vesicles are present in human blood plasma. Int Immunol.

[34] Ogawa Y, Miura Y, Harazono A, Kanai-Azuma M, Akimoto Y, Kawakami H (2011). Proteomic analysis of two types of exosomes in human whole saliva. Biol Pharm Bull.

[35] Admyre C, Johansson SM, Qazi KR, Filén J-J, Lahesmaa R, Norman M (2007). Exosomes with immune modulatory features are present in human breast milk. J Immunol.

[36] Street JM, Barran PE, Mackay CL, Weidt S, Balmforth C, Walsh TS (2012). Identification and proteomic profiling of exosomes in human cerebrospinal fluid. J Transl Med.

[37] Lee Y, El Andaloussi S, Wood MJ (2012). Exosomes and microvesicles: extracellular vesicles for genetic information transfer and gene therapy. Hum Mol Genet.

[38] Sant DW, Mustafi S, Gustafson CB, Chen J, Slingerland JM, Wang G (2018). Vitamin C promotes apoptosis in breast cancer cells by increasing TRAIL expression. Sci Rep.

[39] Matsuzaki K, Fujita K, Jingushi K, Kawashima A, Ujike T, Nagahara A (2017). MiR-21-5p in urinary extracellular vesicles is a novel biomarker of urothelial carcinoma. Oncotarget.

[40] He L, Zhu D, Wang J, Wu X (2019). A highly efficient method for isolating urinary exosomes. Int. J. Mol. Med.

[41] Raimondo F, Morosi L, Corbetta S, Chinello C, Brambilla P, Della Mina P (2013). Differential protein profiling of renal cell carcinoma urinary exosomes. Mol Biosyst.

[42] Salih M, Fenton RA, Knipscheer J, Janssen JW, Vredenbregt-van den Berg MS, Jenster G (2016). An immunoassay for urinary extracellular vesicles. Am J Physiol Renal Physiol.

[43] Liang L-G, Kong M-Q, Zhou S, Sheng Y-F, Wang P, Yu T (2017). An integrated double-filtration microfluidic device for isolation, enrichment and quantification of urinary extracellular vesicles for detection of bladder cancer. Sci Rep.

[44] Murakami T, Yamamoto CM, Akino T, Tanaka H, Fukuzawa N, Suzuki H (2018). Bladder cancer detection by urinary extracellular vesicle mRNA analysis. Oncotarget.

[45] Motamedinia P, Scott AN, Bate KL, Sadeghi N, Salazar G, Shapiro E (2016). Urine exosomes for non-invasive assessment of gene expression and mutations of prostate cancer. PLoS One.

[46] Zhou X, Mao A, Wang X, Duan X, Yao Y, Zhang C (2013). Urine and serum microRNA-1 as novel biomarkers for myocardial injury in open-heart surgeries with cardiopulmonary bypass. PloS one.

[47] Cheng Y, Wang X, Yang J, Duan X, Yao Y, Shi X (2012). A translational study of urine miRNAs in acute myocardial infarction. J Mol Cell Cardiol.

[48] Shang J-W, Yan X-L, Zhang H, Su S-B (2019). Expression and significance of urinary microRNA in patients with chronic hepatitis B. Medicine.

[49] Yang X, Greenhaw J, Shi Q, Su Z, Qian F, Davis K (2012). Identification of urinary microRNA profiles in rats that may diagnose hepatotoxicity. Toxicol Sci.

[50] Alvarez ML, Khosroheidari M, Ravi RK, DiStefano JK (2012). Comparison of protein, microRNA, and mRNA yields using different methods of urinary exosome isolation for the discovery of kidney disease biomarkers. Kidney Int.

[51] Musante L, Saraswat M, Ravidà A, Byrne B, Holthofer H (2013). Recovery of urinary nanovesicles from ultracentrifugation supernatants. Nephrol Dial Transplant.

[52] Kesson A, Talbott J, Gyory A (1978). Microscopic examination of urine. The Lancet.

[53] Wilson LA (2005). Urinalysis. Nurs Stand.

[54] Soubra A, Risk MC (2015). Diagnostics techniques in non-muscle invasive bladder cancer. Indian journal of urology: IJU: Indian J Urol.

[55] Cwiklinska A, Kakol J, Kuchta A, Kortas-Stempak B, Pacanis A, Rogulski J (2012). The standardization of urine particle counting in medical laboratories-a Polish experience with the EQA programme. Scand J Clin Lab Invest.

[56] Schade GR, Faerber GJ (2010). Urinary tract stones. Primary Care: Clinics in Office Practice.

[57] Andersson E, Dahmcke CM, Steven K, Larsen LK, Guldberg P (2015). Filtration Device for On-Site Collection, Storage and Shipment of Cells from Urine and Its Application to DNA-Based Detection of Bladder Cancer. PLoS One.

[58] Bacchin P, Derekx Q, Veyret D, Glucina K, Moulin P (2013). Clogging of microporous channels networks: role of connectivity and tortuosity. Microfluid Nanofluidics.

[59] Birkhahn M, Mitra AP, Williams AJ, Barr NJ, Skinner EC, Stein JP (2013). A novel precision-engineered microfiltration device for capture and characterisation of bladder cancer cells in urine. Eur J Cancer.

[60] He L, Zhu D, Wang J, Wu X (2019). A highly efficient method for isolating urinary exosomes. Int J Mol Med.

[61] Kuntaegowdanahalli SS, Bhagat AA, Kumar G, Papautsky I (2009). Inertial microfluidics for continuous particle separation in spiral microchannels. Lab Chip.

[62] He H, Han C, Hao L, Zang G (2016). ImmunoCyt test compared to cytology in the diagnosis of bladder cancer: A meta-analysis. Oncol Lett.

[63] Comploj E, Mian C, Ambrosini-Spaltro A, Dechet C, Palermo S, Trenti E (2013). uCyt+/ImmunoCyt and cytology in the detection of urothelial carcinoma: an update on 7422 analyses. Cancer Cytopathol.

[64] Sinclair A, Xie X, Dendukuri N The clinical effectiveness and cost of a pneumococcal urine antigen immunochromatographic test (BinaxNOW Streptococcus pneumoniae) in the diagnosis of community acquired Streptococcus pneumoniae pneumonia in patients admitted to hospital: Technology Assessment Unit of the McGill University Health Centre. 2012.

[65] Domanguez J, Gal N, Blanco S, Pedroso P, Prat C, Matas L (2001). Detection of Streptococcus pneumoniae antigen by a rapid immunochromatographic assay in urine samples. Chest.

[66] Grossman HB, Messing E, Soloway M, Tomera K, Katz G, Berger Y (2005). Detection of bladder cancer using a point-of-care proteomic assay. Jama.

[67] Moonen P, Kiemeney L, Witjes J (2005). Urinary NMP22® BladderChek® test in the diagnosis of superficial bladder cancer. Eur Urol.

[68] Cheng Z-Z, Corey MJ, Parepalo M, Majno S, Hellwage J, Zipfel PF (2005). Complement factor H as a marker for detection of bladder cancer. Clin Chem.

[69] Irani J, Desgrandchamps F, Millet C, Toubert M-E, Bon D, Aubert J (1999). BTA stat and BTA TRAK: A comparative evaluation of urine testing for the diagnosis of transitional cell carcinoma of the bladder. Eur Urol.

[70] Thomas L, Leyh H, Marberger M, Bombardieri E, Bassi P, Pagano F (1999). Multicenter trial of the quantitative BTA TRAK assay in the detection of bladder cancer. Clin Chem.

[71] Baños JLG, Rodrigo MdHR, Juárez FMA, García BM (2001). Usefulness of the BTA STAT Test for the diagnosis of bladder cancer. Urology.

[72] Miyake M, Goodison S, Rizwani W, Ross S, Grossman HB, Rosser CJ (2012). Urinary BTA: indicator of bladder cancer or of hematuria. World J Urol.

[73] Meeting WECoBS WHO Expert Committee on Biological Standardization: Sixtieth Report: World Health Organization. 2013.

[74] Henriques-Normark B, Tuomanen EI (2013). The pneumococcus: epidemiology, microbiology, and pathogenesis. Cold Spring Harb Perspect Med.

[75] Rosser CJ, Chang M, Dai Y, Ross S, Mengual L, Alcaraz A (2014). Urinary protein biomarker panel for the detection of recurrent bladder cancer. Cancer Epidemiol Biomarkers Prev.

[76] Kumar P, Nandi S, Tan TZ, Ler SG, Chia KS, Lim W-Y (2015). Highly sensitive and specific novel biomarkers for the diagnosis of transitional bladder carcinoma. Oncotarget.

[77] Sokolova IA, Halling KC, Jenkins RB, Burkhardt HM, Meyer RG, Seelig SA (2000). The development of a multitarget, multicolor fluorescence in situ hybridization assay for the detection of urothelial carcinoma in urine. J Mol Diagn.

[78] Kipp BR, Halling KC, Campion MB, Wendel AJ, Karnes RJ, Zhang J (2008). Assessing the value of reflex fluorescence in situ hybridization testing in the diagnosis of bladder cancer when routine urine cytological examination is equivocal. J Urol.

[79] Hajdinjak T (2008). UroVysion FISH test for detecting urothelial cancers: meta-analysis of diagnostic accuracy and comparison with urinary cytology testing. Urol Oncol: Elsevier.

[80] Lavery HJ, Zaharieva B, McFaddin A, Heerema N, Pohar KS (2017). A prospective comparison of UroVysion FISH and urine cytology in bladder cancer detection. BMC cancer.

[81] Thompson IM, Pauler DK, Goodman PJ, Tangen CM, Lucia MS, Parnes HL (2004). Prevalence of prostate cancer among men with a prostate-specific antigen level≤ 4.0 ng per milliliter. N Engl J Med.

[82] Roobol MJ, Schröder FH, van Leeuwen P, Wolters T, van den Bergh RC, van Leenders GJ (2010). Performance of the prostate cancer antigen 3 (PCA3) gene and prostate-specific antigen in prescreened men: exploring the value of PCA3 for a first-line diagnostic test. Eur Urol.

[83] Groskopf J, Aubin SM, Deras IL, Blase A, Bodrug S, Clark C (2006). APTIMA PCA3 molecular urine test: development of a method to aid in the diagnosis of prostate cancer. Clin Chem.

[84] Urquidi V, Netherton M, Gomes-Giacoia E, Serie D, Eckel-Passow J, Rosser CJ (2016). Urinary mRNA biomarker panel for the detection of urothelial carcinoma. Oncotarget.

[85] Mengual L, Lozano JJ, Ingelmo-Torres M, Izquierdo L, Musquera M, Ribal MJ (2016). Using gene expression from urine sediment to diagnose prostate cancer: development of a new multiplex mRNA urine test and validation of current biomarkers. BMC cancer.

[86] Matsumura Y, Hanbury D, Smith J, Tarin D (1994). Non-invasive detection of malignancy by identification of unusual CD44 gene activity in exfoliated cancer cells. BMJ.

[87] Descotes F, Kara N, Decaussin-Petrucci M, Piaton E, Geiguer F, Rodriguez-Lafrasse C (2017). Non-invasive prediction of recurrence in bladder cancer by detecting somatic TERT promoter mutations in urine. Br J Cancer.

[88] Vogelstein B, Kinzler KW (1999). Digital pcr. Proc Natl Acad Sci U S A.

[89] Perkins G, Lu H, Garlan F, Taly V (2017). Droplet-based digital PCR: application in cancer research. Adv Clin Chem: Elsevier.

[90] Birkenkamp-Demtröder K, Nordentoft I, Christensen E, Høyer S, Reinert T, Vang S (2016). Genomic alterations in liquid biopsies from patients with bladder cancer. Eur Urol.

[91] Nakano M, Komatsu J, Matsuura S-i, Takashima K, Katsura S, Mizuno A (2003). Single-molecule PCR using water-in-oil emulsion. J Biotechnol.

[92] Hindson BJ, Ness KD, Masquelier DA, Belgrader P, Heredia NJ, Makarewicz AJ (2011). High-throughput droplet digital PCR system for absolute quantitation of DNA copy number. Anal Chem.

[93] Lodewijk I DM, Rubio C, Munera-Maravilla E, Segovia C, Bernardini A, Teijeira A, Paramio JM, Suárez-Cabrera C (2018). Liquid Biopsy Biomarkers in Bladder Cancer: A Current Need for Patient Diagnosis and Monitoring. Int J Mol Sci.

[94] Dressman D, Yan H, Traverso G, Kinzler KW, Vogelstein B (2003). Transforming single DNA molecules into fluorescent magnetic particles for detection and enumeration of genetic variations. Proc Natl Acad Sci U S A.

[95] Millholland JM, Li S, Fernandez CA, Shuber AP (2012). Detection of low frequency FGFR3 mutations in the urine of bladder cancer patients using next-generation deep sequencing. Res Rep Urol.

[96] Ansorge WJ (2009). Next-generation DNA sequencing techniques. N Biotechnol.

[97] Eich M-L, Pena MDCR, Springer SU, Taheri D, Tregnago AC, Salles DC (2019). Incidence and distribution of UroSEEK gene panel in a multi-institutional cohort of bladder urothelial carcinoma. Mod Pathol.

[98] Springer SU, Chen C-H, Pena MDCR, Li L, Douville C, Wang Y (2018). Non-invasive detection of urothelial cancer through the analysis of driver gene mutations and aneuploidy. Elife.

[99] Dudley JC, Schroers-Martin J, Lazzareschi DV, Shi WY, Chen SB, Esfahani MS (2019). Detection and surveillance of bladder cancer using urine tumor DNA. Cancer Discov.

[100] Shekhtman EM, Anne K, Melkonyan HS, Robbins DJ, Warsof SL, Umansky SR (2009). Optimization of transrenal DNA analysis: detection of fetal DNA in maternal urine. Clin Chem.

[101] Strasinger SK, Di Lorenzo M, Di Lorenzo MS (2014). Urinalysis and Body Fluids. Philadelphia: F.A. Davis.

[102] Smith GT, Dwork N, Khan SA, Millet M, Magar K, Javanmard M (2016). Robust dipstick urinalysis using a low-cost, micro-volume slipping manifold and mobile phone platform. Lab chip.

[103] Kutter D (2000). The urine test strip of the future. Clin Chim Acta.

[104] Organization WH (2019). The WHO global task force on TB impact measurement. World Health Organization.

[105] Correia-Neves M, Froberg G, Korshun L, Viegas S, Vaz P, Ramanlal N (2019). Biomarkers for tuberculosis: the case for lipoarabinomannan. ERJ Open Res.

[106] Reither K, Saathoff E, Jung J, Minja LT, Kroidl I, Saad E (2009). Low sensitivity of a urine LAM-ELISA in the diagnosis of pulmonary tuberculosis. BMC Infect Dis.

[107] Murray RW Precipitation (chemistry). 2019.

[108] Hilbrig F (2003). Protein purification by affinity precipitation. J Chromatogr B Analyt Technol Biomed Life Sci.

[109] Gossett DR, Weaver WM, Mach AJ, Hur SC, Tse HT, Lee W (2010). Label-free cell separation and sorting in microfluidic systems. Anal Bioanal Chem.

[110] Collins DJ, Khoo BL, Ma Z, Winkler A, Weser R, Schmidt H (2017). Selective particle and cell capture in a continuous flow using micro-vortex acoustic streaming. Lab Chip.

[111] Zborowski M, Ostera GR, Moore LR, Milliron S, Chalmers JJ, Schechter AN (2003). Red blood cell magnetophoresis. Biophys J.

[112] Huh D, Bahng JH, Ling Y, Wei H-H, Kripfgans OD, Fowlkes JB (2007). Gravity-driven microfluidic particle sorting device with hydrodynamic separation amplification. Anal Chem.

[113] Di Carlo D, Irimia D, Tompkins RG, Toner M (2007). Continuous inertial focusing, ordering, and separation of particles in microchannels. Proc Natl Acad Sci U S A.

[114] Yu ZT, Aw Yong KM, Fu J (2014). Microfluidic blood cell sorting: now and beyond. Small.

[115] Khoo BL, Bouquerel C, Durai P, Anil S, Goh B, Wu B (2019). Detection of Clinical Mesenchymal Cancer Cells from Bladder Wash Urine for Real-Time Detection and Prognosis. Cancers (Basel).

[116] Warkiani ME, Khoo BL, Wu L, Tay AK, Bhagat AA, Han J (2016). Ultra-fast, label-free isolation of circulating tumor cells from blood using spiral microfluidics. Nat Protoc.

[117] Peng Zhang XZ, Mei He, Yuqin Shang, Ashley L (2019). Tetlow, Andrew K. Godwin, Yong Zeng Ultrasensitive detection of circulating exosomes with a 3D-nanopatterned microfluidic chip. Nat Biomed Eng.

[118] Chen GD FF, Fernandez-Suarez M, Wardle BL, Toner M (2011). Nanoporous elements in microfluidics for multi-scale manipulation of bioparticles. Small.

[119] Grace D (2014). Chen FFEC, Brian L, Toner M. Nanoporous micro-element arrays for particle interception in microfluidic cell separation. Lab Chip.

[120] Abellan-Llobregat A, Gonzalez-Gaitan C, Vidal L, Canals A, Morallon E (2018). Portable electrochemical sensor based on 4-aminobenzoic acid-functionalized herringbone carbon nanotubes for the determination of ascorbic acid and uric acid in human fluids. Biosens Bioelectron.

[121] Wunsch BH, Smith JT, Gifford SM, Wang C, Brink M, Bruce RL (2016). Nanoscale lateral displacement arrays for the separation of exosomes and colloids down to 20 nm. Nat Nanotechnol.

[122] Davis JA ID, Morton KJ, Lawrence DA, Huang LR, Chou SY, Sturm JC, Austin RH (2006). Deterministic hydrodynamics: taking blood apart. Proc Natl Acad Sci U S A.

[123] Wunsch BH, Smith JT, Gifford SM, Wang C, Brink M, Bruce RL (2016). Nanoscale lateral displacement arrays for the separation of exosomes and colloids down to 20 nm. Nat Nanotechnol.

[124] Huang LR, Cox EC, Austin RH, Sturm JC (2004). Continuous particle separation through deterministic lateral displacement. Science.

[125] Zheng G, Patolsky F, Cui Y, Wang WU, Lieber CM (2005). Multiplexed electrical detection of cancer markers with nanowire sensor arrays. Nat Biotechnol.

[126] Yasui T, Yanagida T, Ito S, Konakade Y, Takeshita D, Naganawa T (2017). Unveiling massive numbers of cancer-related urinary-microRNA candidates via nanowires. Sci Adv.

[127] Chiriaco MS, Bianco M, Nigro A, Primiceri E, Ferrara F, Romano A (2018). Lab-on-Chip for Exosomes and Microvesicles Detection and Characterization. Sensors (Basel).

[128] Liang L, Wang Y, Lu S, Kong M, Lin Y, Cuzzucoli F (2018). Microchips for detection of exfoliated tumor cells in urine for identification of bladder cancer. Anal Chim Acta.

[129] Bhagat AA, Kuntaegowdanahalli SS, Papautsky I (2008). Continuous particle separation in spiral microchannels using Dean flows and differential migration. Lab Chip.

[130] Lyu Y, Cui D, Huang J, Fan W, Miao Y, Pu K (2019). Near-Infrared Afterglow Semiconducting Nano-Polycomplexes for the Multiplex Differentiation of Cancer Exosomes. Angew Chem Int Ed Engl.

[131] Li Y, Gecevicius M, Qiu J (2016). Long persistent phosphors-from fundamentals to applications. Chem Soc Rev.

[132] Wang Y, Li Z, Lin Q, Wei Y, Wang J, Li Y (2019). Highly Sensitive Detection of Bladder Cancer-Related miRNA in Urine Using Time-Gated Luminescent Biochip. ACS Sens.

[133] Choi HS, Liu W, Misra P, Tanaka E, Zimmer JP, Itty Ipe B (2007). Renal clearance of quantum dots. Nat Biotechnol.

[134] Loynachan CN, Soleimany AP, Dudani JS, Lin Y, Najer A, Bekdemir A (2019). Renal clearable catalytic gold nanoclusters for in vivo disease monitoring. Nat Nanotechnol.

[135] Liu C, Hu G (2017). High-Throughput Particle Manipulation Based on Hydrodynamic Effects in Microchannels. Micromachines (Basel).

[136] Gou Y, Jia Y, Wang P, Sun C (2018). Progress of Inertial Microfluidics in Principle and Application. Sensors (Basel).

[137] Martel JM, Toner M (2014). Inertial focusing in microfluidics. Annu Rev Biomed Eng.

[138] Khoo BL, Shang M, Ng CH, Lim CT, Chng WJ, Han J (2019). Liquid biopsy for minimal residual disease detection in leukemia using a portable blast cell biochip. NPJ Precis Oncol.

[139] Tay HM, Kharel S, Dalan R, Chen ZJ, Tan KK, Boehm BO (2017). Rapid purification of sub-micrometer particles for enhanced drug release and microvesicles isolation. NPG Asia Materials.

[140] Ra M, Muhammad MS, Lim C, Han S, Jung C, Kim WY (2018). Smartphone-Based Point-of-Care Urinalysis Under Variable Illumination. IEEE J Transl Eng Health Med.

[141] Jalal UM, Jin GJ, Shim JS (2017). Paper-Plastic Hybrid Microfluidic Device for Smartphone-Based Colorimetric Analysis of Urine. Anal Chem.

[142] Karlsen H, Dong T (2017). Smartphone-Based Rapid Screening of Urinary Biomarkers. IEEE Trans Biomed Circuits Syst.

[143] Oncescu V, O'Dell D, Erickson D (2013). Smartphone based health accessory for colorimetric detection of biomarkers in sweat and saliva. Lab Chip.

[144] Malekjahani A, Sindhwani S, Syed AM, Chan WCW (2019). Engineering Steps for Mobile Point-of-Care Diagnostic Devices. Acc Chem Res.

[145] Diehl F, Schmidt K, Choti MA, Romans K, Goodman S, Li M (2008). Circulating mutant DNA to assess tumor dynamics. Nat Med.

[146] Xia Y, Huang CC, Dittmar R, Du M, Wang Y, Liu H (2016). Copy number variations in urine cell free DNA as biomarkers in advanced prostate cancer. Oncotarget.

[147] Bettegowda C, Sausen M, Leary RJ, Kinde I, Wang Y, Agrawal N (2014). Detection of Circulating Tumor DNA in Early- and Late-Stage Human Malignancues. Sci Transi Med.

[148] Utting M, Werner W, Dahse R, Schubert J, Junker K (2002). Microsatellite analysis of free tumor DNA in urine, serum, and plasma of patients: a minimally invasive method for the detection of bladder cancer. Clin Cancer Res.

[149] Casadio V CD, Salvi S, Gunelli R, Carretta E, Amadori D, Silvestrini R, Zoli W (2013). Urine cell-free DNA integrity as a marker for early prostate cancer diagnosis: a pilot study. Biomed Res Int. 2013.

[150] Clayton A, Harris CL, Court J, Mason MD, Morgan BP (2003). Antigen-presenting cell exosomes are protected from complement-mediated lysis by expression of CD55 and CD59. Eur J Immunol.

[151] Guescini M, Genedani S, Stocchi V, Agnati LF (2010). Astrocytes and Glioblastoma cells release exosomes carrying mtRNA. J Neural Transm (Vienna).

[152] Marzesco AM, Janich P, Wilsch-Bräuninger M, Dubreuil V, Langenfeld K, Corbeil D (2005). et al. Release of extracellular membrane particles carrying the stem cell marker prominin-1 (CD133) from neural progenitors and other epithelial cells. J Cell Sci.

[153] Raposo G, Nijman HW, Stoorvogel W, Liejendekker R, Harding CV, Melief CJ (1996). B lymphocytes secrete antigen-presenting vesicles. J Exp Med.

[154] Colombo M, Moita C, van Niel G, Kowal J, Vigneron J, Benaroch P (2013). Analysis of ESCRT functions in exosome biogenesis, composition and secretion highlights the heterogeneity of extracellular vesicles. J Cell Sci.

[155] Kowal J, Arras G, Colombo M, Jouve M, Morath JP, Primdal-Bengtson B (2016). Proteomic comparison defines novel markers to characterize heterogeneous populations of extracellular vesicle subtypes. Proc Natl Acad Sci U S A.

